# Sex differences in adults with ADHD: A population based study

**DOI:** 10.1192/j.eurpsy.2025.426

**Published:** 2025-08-26

**Authors:** F. M. Ferrés, C. F. Grau, S. A. Guadall, V. R. Fernández, M. C. De La Cruz, J. A. R. Quiroga

**Affiliations:** 1 Psychiatry, Vall d’Hebron, Barcelona, Spain

## Abstract

**Introduction:**

Attention-deficit/hyperactivity disorder (ADHD) is a common neurodevelopmental disorder that often persists into adulthood, significantly impacting daily functioning and quality of life. Studying sex differences in ADHD is crucial as females are frequently underdiagnosed or misdiagnosed, which can delay treatment and worsen outcomes. ADHD presents in three main subtypes: inattentive, hyperactive-impulsive, and combined. The combined subtype tends to cause more significant impairments, particularly in academic and social contexts. Males are more likely to be diagnosed with hyperactive-impulsive or combined types, while females often present with the inattentive subtype. A subtype-specific approach is essential, as it guides targeted interventions to address distinct behavioral and cognitive challenges, enhancing treatment efficacy and outcomes.

**Objectives:**

This study aims to analyze differences in ADHD severity, comorbidity with other conditions, and socio-functional impact by ADHD subtype and sex, as well as to evaluate interactions between these variables.

**Methods:**

This population-based study included 900 adults diagnosed with ADHD from a specialized ADHD clinic. Participants were classified by ADHD subtype and sex. Diagnostic and severity assessments were conducted using validated tools, including the CAADID-I, DIVA-5, ADHD Rating Scale (ADHD-RS), Wender Utah Rating Scale (WURS), and Clinical Global Impression Severity Scale (CGI-S). Comorbid psychiatric conditions and psychosocial functioning were evaluated using the BDI-II, STAI, BIS-11, PSQI, FAST, and WHODAS scales. Statistical analyses included bivariate, multivariate, and General Linear Model (GLM) methods.

**Results:**

Females were diagnosed with ADHD later than males (p=0.001) and exhibited greater severity (ADHD-RS, p<0.001) and higher levels of depression and anxiety. No significant sex differences were observed in impulsivity or sleep quality. The combined ADHD subtype was associated with greater clinical severity and functional impairment. An interaction effect was found between sex and ADHD subtype only for WHODAS scores, with females in the combined subtype showing greater impairment.

**Conclusions:**

ADHD presents differently across sexes and subtypes, with specific interactions observed in functional impairment. These findings emphasize the importance of considering sex and ADHD subtype independently to enhance diagnostic accuracy and inform targeted treatment strategies.

**Disclosure of Interest:**

None Declared

